# Setting priorities for healthcare interventions in Indonesia: a comprehensive conceptual framework

**DOI:** 10.1186/s12939-025-02668-z

**Published:** 2025-11-24

**Authors:** Mohammed Alfaqeeh, Neily Zakiyah, Maarten J. Postma, Auliya A. Suwantika

**Affiliations:** 1 Department of Pharmacology and Clinical Pharmacy, Faculty of Pharmacy, Universitas Padjadjaran, Bandung, 45363 Indonesia; 2 Center of Excellence for Pharmaceutical Care Innovation, Universitas Padjadjaran, Bandung, Indonesia; 3 Department of Health Sciences, University Medical Center Groningen, University of Groningen, Groningen, The Netherlands; 4 Department of Economics, Econometrics and Finance, Faculty of Economics and Business, University of Groningen, Groningen, The Netherlands; 5 Center for Health Technology Assessment, Universitas Padjadjaran, Bandung, Indonesia

**Keywords:** Healthcare access, Health outcomes, Resource allocation, Decision-making, Policy implications

## Abstract

Health priority setting is a fundamental aspect of public health decision-making in Indonesia, a country characterized by its vast geographic diversity, large population, and evolving healthcare challenges. Given the complex and varied health needs across the country, effective prioritization of healthcare interventions is essential for optimizing resource allocation and improving health outcomes. This study aims to explore the current healthcare priority-setting strategies in Indonesia and identify opportunities to enhance their implementation to support the achievement of universal health coverage (UHC). We conducted a narrative review of relevant literature using the medical databases PubMed and EMBASE, supplemented by a manual review of reference lists and key policy documents. Key findings reveal that the criteria for health intervention prioritization in Indonesia include disease burden, intervention effectiveness, cost, acceptability, and fairness. However, challenges persist in the consistent application of these criteria across Indonesia’s diverse regions, further compounded by disparities in infrastructure, governance, and data availability. In terms of healthcare priority setting, Indonesia combines technical approaches like Health Technology Assessment and Programme Budgeting and Marginal Analysis with value-based methods such as the Delphi Technique and Multi-Criteria Decision Analysis, focusing on equity, fairness, and stakeholder engagement. Limitations in current approaches for healthcare priority setting further complicate the process. Additionally, ethical and social considerations sometimes conflict with economic or technical priorities, underscoring the need for continuous evaluation and adaptation of health priority setting practices. These complexities highlight that while Indonesia has made significant strides, areas for improvement remain to ensure equitable and effective healthcare allocation. Using Indonesia as a reference case, this paper emphasizes the importance of integrating evidence-based priority setting within the framework of UHC to improve healthcare access and equity in Indonesia. This analysis equips stakeholders with the knowledge necessary to navigate Indonesia’s complex healthcare landscape and drive initiatives aimed at achieving better health outcomes and the well-being of its population.

## Introduction

Priority setting refers to the process of making deliberate choices about which health services, programs, or population needs should be given precedence in the allocation of limited resources [1]. In healthcare, priority setting serves as a roadmap in the complex landscape of public health, illuminating the path toward optimal resource allocation and improved health outcomes [2]. Governments typically prioritize health spending to maximize value for money by addressing market failures, improving efficiency, and achieving better population outcomes [3]. Essential services encompass both clinical care, such as diagnosis and treatment, and public health interventions, such as immunization, all of which require careful resource allocation [4]. However, determining these priorities remains a persistent challenge globally, especially as countries work to balance resource constraints with diverse health needs [5]. Effective prioritization is becoming more critical as populations grow, demands for improved health increase, technical advancements in addressing health issues expand, and available resources become more constrained, especially for many low- and middle-income countries (LMICs) [6]. These countries face the challenges of the epidemiological transition, where they experience both the burden of infectious diseases and the rising prevalence of non-communicable diseases (NCDs), a situation often referred to as the dual burden of disease [7, 8].

Indonesia, the fourth most populous country in the world, is categorized by the World Bank as an upper middle-income country, with a Gross National Income (GNI) per capita of approximately USD 4,870 (2023) [9]. With its sprawling archipelago comprising over 17,000 islands and a population exceeding 270 million [10]. Indonesia deals with various health challenges ranging from infectious diseases to increasing non-communicable conditions [11, 12]. The Indonesian government has established the National Medium-Term Development Plan for the Health Sector 2020–2024 to enhance health services toward Universal Health Coverage (UHC). This plan bolsters primary healthcare through preventive measures, innovation, and technology utilization [13]. Additionally, the Indonesian Ministry of Health (MOH) has outlined five strategic health development priorities for the next five years, including maternal and child health, reproductive health, community nutrition enhancement, health system reinforcement, and drug and food supervision [14]. Furthermore, the Indonesian MOH (2020) actively addresses concerns such as stunting, maternal and infant mortality rates, national health insurance management enhancement, healthcare service reinforcement, drug quality assurance, and medical device self-sufficiency [15]. Despite these remarkable efforts in improving healthcare access and delivery, Indonesia faces persistent disparities in health outcomes across regions and demographic groups [16, 17].

Healthcare priority setting in Indonesia is a multi-faceted process led by key agencies such as the MOH, the National Development Planning Agency (Bappenas), and the Social Security Agency for Health (BPJS), with significant input from local health authorities, research institutions, and Parliament [18–20]. The MOH sets national health policies and coordinates priorities across sectors [21]. while Bappenas integrates healthcare into broader national development plans [22]. Social Security Agency for Health (BPJS Kesehatan) manages the national health insurance system, ensuring equitable access to essential services, and local authorities tailor policies to regional needs [23]. The National Commission on Healthcare Ethics and Economics advises on resource allocation by incorporating ethical, economic, and social considerations, while research institutes provide evidence-based recommendations for effective interventions [24]. Parliament plays an oversight role, reviewing healthcare budgets and advocating for reforms [25]. This collaborative approach, which combines top-down policymaking with bottom-up local adaptation, is essential in addressing Indonesia’s diverse health challenges, although resource limitations and regional disparities remain ongoing obstacles to achieving equitable and effective healthcare across the country.

In the context of Indonesia’s evolving healthcare landscape, addressing health priorities requires a comprehensive strategy that considers not only the country’s epidemiological trends but also its resource constraints, cultural factors, and societal values. This review aims to assess the current state of healthcare priority-setting strategies in Indonesia and to identify opportunities for improving their implementation in support of achieving UHC.

## Methods

A nonsystematic literature review was conducted to explore healthcare priority-setting strategies in Indonesia. Peer-reviewed literature was identified through searches in PubMed and EMBASE using combinations of the following keywords: healthcare priority setting, Indonesia health system, universal health coverage, health interventions, health technology assessment, national health insurance (*Jaminan Kesehatan Nasional, JKN*), health policy, and related terms. Additionally, reference lists of retrieved articles were manually reviewed to capture additional sources that were not identified in the database search.

In addition to peer-reviewed studies, we included key policy documents and strategic reports issued by the Indonesian MOH, Bappenas, and BPJS Kesehatan. Documents such as the National Medium-Term Development Plan, Strategic Plans (Renstra), and JKN-related reports were reviewed. We also incorporated relevant international frameworks and guidance, including those from the World Health Organization (WHO), to compare Indonesian practices with global standards. The findings were summarized, and recommendations were outlined.

## Criteria for health intervention prioritization

**Fig. 1:**
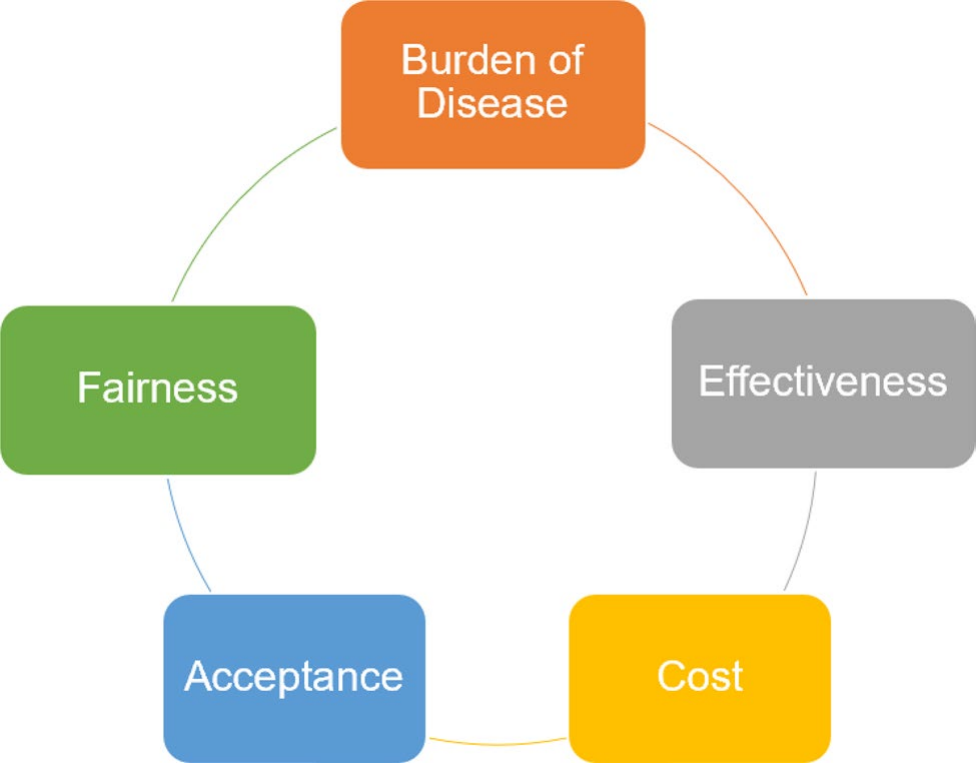
WHO criteria for health intervention Prioritization. Source: adapted from chapter 4 of Strategizing National health in the twenty-first Century: a handbook (World health Organization, 2016) [28]

Prioritizing health interventions is a critical aspect of healthcare planning and resource allocation [26]. Various criteria are typically considered when determining the priority of health interventions. These criteria assist decision-makers in efficiently and effectively directing resources to address a population’s most urgent health needs. According to the WHO, national health priority setting should be guided by five core criteria: burden of disease, effectiveness, cost, acceptability, and fairness of an intervention [27]. These criteria are intended to guide priority-setting processes in support of achieving Universal Health Coverage (UHC), while allowing flexibility for contextual adaptation. These criteria are interconnected and should be considered in a comprehensive, balanced manner when making healthcare prioritization decisions (Fig. 1) [28]. Several approaches have been developed to assess healthcare priorities based on these criteria. Some focus on a single criterion such as cost-effectiveness or disease burden [29, 30], while others, like health technology assessment (HTA) [31], typically integrate multiple criteria to support more comprehensive evaluations. The choice between single- or multi-criterion approaches often depends on the specific policy question or decision-making context [32].

Adapting WHO’s priority-setting frameworks is crucial for Indonesia’s unique healthcare landscape, which faces both communicable and NCDs and operates within a decentralized health system [33]. Moreover, Indonesia’s diverse cultural norms, beliefs, and practices influence healthcare perceptions and access differently across urban and rural areas [34]. While Indonesia has not formally adopted a standardized framework explicitly based on all five WHO-recommended criteria for healthcare prioritization, several national policies and guidelines incorporate similar principles [35]. For instance, the Ministry of Health’s strategic plans (*Rencana Strategis Kementerian Kesehatan*) and National Health Insurance (*Jaminan Kesehatan Nasional*, JKN) policies reflect explicit considerations of disease burden, cost-effectiveness, and equity in determining healthcare priorities [36, 37]. Additionally, HTA has been increasingly used to inform decision-making, particularly for the inclusion of interventions in JKN coverage [38]. However, the criteria of acceptability and fairness, while acknowledged in some policy discussions, are not yet consistently or systematically integrated into formal frameworks. The extent to which each criterion is weighted or systematically applied in decision-making remains an area requiring further clarification and formalization. By incorporating WHO-recommended priority-setting guidelines tailored to Indonesia’s specific needs, policymakers can make informed decisions to enhance the health system’s efficiency, equity, and effectiveness, ultimately improving health outcomes for all Indonesians.

### Burden of disease

The burden of the health issue is a quantitative parameter based on time, measured by considering the time lost due to illness or death from a disease [39]. It involves a comprehensive assessment of the physical and psychosocial health impacts of diseases, conditions, and associated risk factors [40]. Assessing the disease burden in Indonesia involves various indicators tailored to the country’s multifaceted nature of public health. These indicators encompass a wide range of measures, including life expectancy, cause-specific mortality rates, incidence and prevalence of specific diseases, perceived health status, occurrences of physical and mental limitations and disabilities, as well as indirect measures such as absenteeism, work incapacity, and healthcare utilization and associated costs [41]. However, it’s important to note that each indicator only captures a singular aspect of burden of disease from a public health’s perspective, focusing either on mortality or morbidity [42]. In Indonesia, efforts to measure the burden of disease have seen some progress, though challenges remain. The Global Burden of Disease (GBD) study has been instrumental in providing detailed estimates for Indonesia, primarily drawing on a combination of national health surveys, hospital records, and regional studies, while also incorporating modeled estimates where direct data is limited [43, 44]. However, there remains a need for more localized data to accurately reflect the diverse health challenges across different regions and populations in Indonesia. The Indonesian government has also adopted several health indicators, such as Disability-adjusted life years (DALYs), to inform policy decisions [45]. DALYs have been used in prioritizing disease control programs, allocating healthcare resources, and guiding investments in public health interventions [46]. For instance, Indonesia has utilized DALY estimates to shape its NCD control strategies [47], determine vaccination priorities [48], and assess the cost-effectiveness of health interventions under the JKN [36].

The burden of a health issue varies across different population groups and is influenced by the proportion at risk of morbidity and mortality [49]. Identifying high-risk groups and evaluating intervention effectiveness are crucial steps. Methodological advancements have led to the development of summary measures of population health (SMPH) [50], integrating both morbidity and mortality data. SMPHs are divided into two categories: health expectancies (e.g., Disability-Free Life Expectancy (DFLE), Healthy Life Years (HLY), and Disability-Adjusted Life Expectancy (DALE)) and health gaps (e.g., DALYs).

DALYs, a key metric from the GBD projects [51–53], measure the gap between ideal and actual health by estimating lost healthy life years due to illness, disability, and mortality [54]. For instance, a disease burden of 100 DALYs per 1000 people-year indicates a loss of 100 healthy life years annually per 1000 individuals [55]. The application of DALYs in Indonesia has highlighted significant public health issues, such as the high burden of NCDs like cardiovascular diseases and diabetes [56]. These findings align with the epidemiological transition seen in many developing countries, where NCDs are becoming more prevalent as infectious diseases are controlled. Morbidity is quantified by Years Lived with Disability (YLDs), calculated by the number of new cases, duration, and disability weight [57, 58]. Mortality is measured by Years of Life Lost (YLLs), calculated by the number of deaths and remaining life expectancy at death [59, 60].

The burden of disease can be viewed from various perspectives, such as the healthcare provider’s or societal perspectives [61]. From a healthcare provider’s perspective, the burden is determined by analyzing epidemiological data such as prevalence, incidence, and survival rates. Indonesia faces a shifting epidemiological profile with rising degenerative and NCDs [62], requiring prioritized prevention and treatment efforts [63]. For example, diarrhea and pneumonia are leading causes of death among children under five, prompting targeted prevention programs like initiatives [64].

Incorporating “value” or “preference for health state” in decision-making allows for a comprehensive assessment of the disease burden [65]. Measures like quality-adjusted life years (QALYs) facilitate the comparison of different diseases and interventions, aiding resource allocation to maximize health outcomes [66]. Anchoring health state utility values (HSUVs) on a scale from zero (death) to one (perfect health) helps quantify the impact of various health states, informing prioritization of health interventions and resource allocation to address Indonesia’s most pressing health challenges [67].

### Effectiveness of the intervention

The effectiveness of a health intervention is a critical criterion in the prioritization process, ensuring that resources are allocated to strategies that yield tangible and significant health outcomes [68]. Effectiveness refers to the ability of an intervention to achieve its intended goals and objectives in improving health outcomes for the target population [69]. Health interventions are often intricate, programmatic, and context-specific, requiring evidence of effectiveness that adequately addresses their complexity [70]. Some questions may arise beyond considering the sheer effectiveness criterion [28]:

- How will the chosen prioritization strategy lead to the expected outcomes?
- What alternative interventions are available?
- What are the feasibility considerations of an intervention in specific situations and conditions, i.e. what are the implementation issues?
- How sustainable is the implementation of a new intervention?

Health interventions are evaluated based on their ability to produce desired health outcomes, such as reducing mortality rates, improving quality of life, or preventing the onset of diseases [71]. Robust evidence from clinical trials, epidemiological studies, and systematic reviews is essential in determining the effectiveness of the interventions [72]. However, their efficacy is primarily influenced by the contextual milieu in which they are implemented [73]. Factors such as population demographics, socioeconomic status, cultural beliefs, and healthcare infrastructure wield significant influence, shaping the magnitude of their impact [74, 75].

In Indonesia, assessing the effectiveness of health interventions requires a nuanced understanding of the local context. For example, the implementation of telemedicine during the COVID-19 pandemic showcased the need for adaptation of global technology to local conditions [76]. In this regard, policymakers are faced with two types of situations to analyze the effectiveness of new interventions; either there is no evidence base at the global level, requiring the search for new evidence through scientific studies, or evidence already exists at the global level but effectiveness needs to be verified for its application in the local context [77]. Ultimately, the potential innovative solutions offered by these new interventions must be considered, requiring in-depth and comprehensive HTA to measure the effectiveness of interventions. HTA involves systematically reviewing and appraising clinical evidence to determine the real-world impact of interventions on health outcomes [78]. In the context of Indonesia’s resource limitations, HTA plays a crucial role in ensuring that only cost-effective and clinically beneficial interventions are integrated into the healthcare system [79]. This process prioritizes interventions with proven effectiveness, ensuring their implementation within the national health system.

### Cost of the intervention

Setting priorities for health interventions involves weighing their potential benefits and the resources they require, emphasizing the need for a comprehensive assessment of both advantages and costs to inform evidence-based decision-making. However, a significant need for greater understanding exists regarding the appropriate methodologies for accurately assessing the costs of interventions, particularly in the context of economic evaluations [80]. The cost criterion encompasses both affordability and cost efficiency [81]. Affordability evaluates the financial feasibility of implementing an intervention within existing budget constraints, ensuring it does not impose excessive financial burdens [82]. This involves aligning intervention costs with available financial resources and assessing net accounting costs, cash flow, and budget allocations [83]. It is crucial to evaluate affordability at various stages of the process using three primary financial statements [84]:

- Budget statement: illustrates resource availability throughout the proposal’s lifespan.
- Cash flow statement: outlines the additional cash flow required if the primary option is pursued.
- Funding statement: demonstrates the resources designated for provision from key stakeholders.

Budget Impact Analysis (BIA) has emerged as a contemporary approach for evaluating the affordability of healthcare interventions [85]. Over recent decades, BIA has become increasingly favored as a decision-making tool for policymakers, aiding in assessing the financial feasibility of implementing new healthcare interventions [86, 87]. BIA considers both the size and characteristics of the target population, as well as the differences between current best practices and the proposed new treatment [88]. BIA evidence is often utilized alongside data from cost-effectiveness analysis (CEA) to enhance the evidence base for resource allocation [89]. In conjunction with Indonesian national economic growth data and assessments of the Indonesian government’s revenue-raising ability, BIA contributes valuable insights into the fiscal sustainability of scaling up new interventions and priority-setting decisions. The analysis helped policymakers understand the financial implications of scaling up the program and ensuring its sustainability within the national budget. Furthermore, assessments of immunization programs have utilized BIA to ensure that interventions are affordable and financially sustainable, considering Indonesia’s economic growth and government revenue capabilities [90].

The perspective of an economic evaluation significantly influences which costs are included and how they are prioritized. The societal perspective is the most comprehensive, encompassing all costs and benefits, including direct medical costs, direct non-medical costs, and indirect costs like productivity losses, aiming to capture the total economic impact on society [91]. The healthcare system perspective includes costs borne by the healthcare system, such as hospital and medication costs, excluding non-healthcare costs, to optimize resource allocation within the healthcare sector [92]. The payer perspective focuses on costs incurred by insurers or government programs, such as reimbursement expenses, and is used to assess the financial impact on these entities’ budgets [93]. The patient perspective considers out-of-pocket expenses, transportation, and lost income, highlighting the financial burden on patients and informing policies to reduce their costs [94]. Each perspective tailors the economic evaluation to the specific interests and responsibilities of different stakeholders, ensuring relevant and targeted insights for decision-making.

Indonesia’s efforts to introduce UHC through the JKN program involved extensive cost-efficiency analysis to ensure financial sustainability and equitable healthcare access [95]. This evaluation considered direct medical costs, administrative expenses, and the broader economic impact of JKN, including its effects on productivity and economic growth [96]. Policymakers relied on actuarial studies, national health accounts, and HTA to assess the financial feasibility of expanding coverage [97]. Cost-effectiveness thresholds were used to prioritize interventions, favoring those that provided the highest health benefits per unit of cost [98]. Additionally, BIA were conducted to estimate the long-term fiscal sustainability of JKN [99]. However, challenges persisted in fully capturing the economic benefits of preventive measures, the indirect costs of disease, and regional variations in healthcare expenditures, highlighting areas for future refinement in priority-setting methodologies. Learning from other countries can further enhance Indonesia’s approach to evaluating the cost of health interventions. For instance, Thailand’s Health Intervention and Technology Assessment Program (HITAP) has been effective in conducting economic evaluations to inform health policy decisions [100]. By adopting similar methodologies and leveraging international collaborations, Indonesia can improve its capacity to conduct comprehensive economic evaluations, ensuring more informed and sustainable health policy decisions.

### Acceptability of the intervention

Acceptability is a critical factor in developing, assessing, and integrating healthcare interventions [101]. It measures the suitability of an intervention from the perspectives of those delivering or receiving it [102]. In decision-making, understanding acceptability ensures that interventions are practical and culturally appropriate, which can lead to better adherence and improved outcomes [103]. Although essential, acceptability alone does not guarantee effectiveness of a cost-efficient intervention. Successful implementation relies on the intervention being acceptable to both providers (such as researchers and healthcare professionals) and recipients (such as patients) [104, 105]. Both patients and healthcare providers who find an intervention acceptable are more likely to follow through with treatment recommendations and effectively utilize diagnostic tools, resulting in enhanced clinical outcomes [106, 107]. From the perspective of patients, the acceptability of an intervention hinges on factors like the content, context, and quality of care provided. Moreover, if a health intervention contradicts social and cultural norms, it can impact community acceptance levels and the success of the intervention. In Indonesia, ensuring the acceptability of healthcare interventions involves navigating diverse cultural and religious considerations. For example, during the implementation of the Measles-Rubella (MR) immunization program in 2018, challenges arose due to a fatwa issued by the Indonesian Ulema Council (MUI), which initially deemed the MR vaccine produced by the Serum Institute of India as forbidden due to its pork-derived ingredients. Despite this, MUI permitted its use in Indonesia for various reasons [108]. As the country with the largest Muslim population globally, the primary obstacle to MR immunization was the relatively low level of community acceptance compared to other vaccines, as the community deemed the vaccine containing forbidden ingredients unacceptable [109].

The perspective of healthcare professionals is another crucial factor in the successful implementation process; if they perceive a lack of acceptability in delivering a particular intervention to patients, there’s a risk that they may deviate from the intended implementation by intervention designers, potentially undermining the intervention’s overall effectiveness [110, 111]. Understanding the acceptability of interventions from both patient and provider perspectives helps in tailoring implementation strategies to enhance uptake and adherence.

**Fig. 2:**
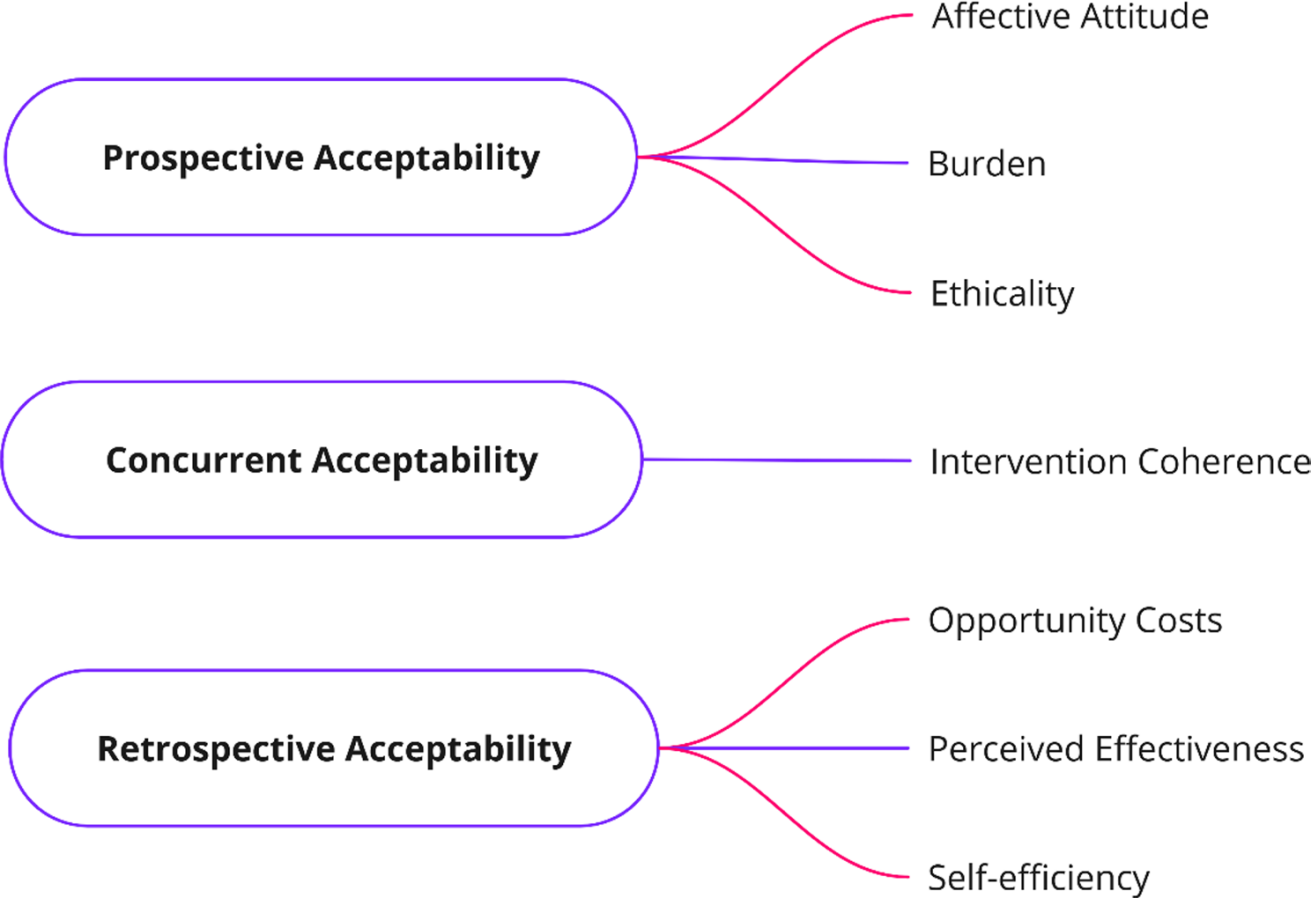
Theoretical Framework of Acceptability (version 2)

The intervention’s acceptability can be assessed from three temporal viewpoints (prospective, concurrent, or retrospective), which vary based on when the evaluation occurs relative to the engagement with the intervention [112]. The Theoretical Framework of Acceptability (TFA) V2 is a revised tool from the TFA V1, used to assess healthcare interventions’ acceptability [101]. It comprises seven components: Affective Attitude, which explores individuals’ emotional responses to the intervention; Burden, assessing perceived effort, time, and inconvenience; Ethicality, evaluating moral appropriateness and alignment with values; Intervention Coherence, examining clarity and logical consistency; Opportunity Costs, measuring perceived sacrifices; Perceived Effectiveness, gauging beliefs in achieving outcomes; and Self-efficacy, reflecting confidence in participation. These components provide insights into different acceptability dimensions, aiding intervention assessment and enhancement strategies (Fig. 2) [101].

### Fairness

Fairness in priority setting can have diverse meanings, ranging from notions of justice and equitable distribution to considerations such as equity in health outcomes, accessibility of healthcare, efficient service management, patient autonomy, and accountability [113]. One important criterion in defining fairness is prioritizing the worse off, as recommended by the WHO Consultative Group on Equity and Universal Health Coverage [114]. This principle emphasizes directing resources toward populations with the greatest health needs, thereby reducing health disparities and promoting more equitable health outcomes [114]. A related concept is the “fair innings” principle, which prioritizes interventions for individuals who have had fewer opportunities to live a full life, favoring younger individuals over those who have already experienced a longer duration of life [115]. The fairness criterion must be considered in setting health program priorities because it is related to the likelihood of trade-offs between an intervention and its effectiveness [116]. A recent example is the COVID-19 pandemic, which had a more significant health impact on Indonesian individuals with low-income levels and not having health insurance, particularly affecting their access to medication and healthcare services [117]. Although Indonesia has experienced high economic growth over the past two decades, this period has also seen a rise in health risks associated with the growth of NCDs [118–120]. It has been reported that prioritizing such interventions is crucial in reducing future health risks and mitigating health inequality in Indonesia [121]. In response, the Indonesian government has implemented efforts to incorporate fairness into priority setting such as the JKN program and equity-focused budgeting. However, the use of fairness metrics in policy decision-making remains limited, and systematic evaluation of their impact across regions is lacking. This highlights the need for further research and the development of transparent, equity-sensitive assessment tools to support fair and consistent priority setting nationwide.

Fairness encompasses various dimensions, including distributive justice, procedural fairness, and ethical considerations. Distributive justice aims to allocate resources based on need, effectiveness, and the potential to improve health outcomes rather than factors such as socioeconomic status or political influence [122]. Procedural fairness involves transparent decision-making processes that involve stakeholders and consider diverse perspectives, ensuring that decisions are made openly, accountably, and with legitimacy [123]. Ethical considerations involve respecting individual rights, autonomy, and dignity, while addressing systemic inequalities and disparities [124]. Another concept linked to fairness is “the rule of rescue”. The rule of rescue entails prioritizing life-saving treatments or procedures, even if it means withholding these resources from others who could benefit from their quality-of-life enhancements [125]. This tension highlights the need to balance fairness in addressing individual cases with efficiency in allocating resources to maximize overall health outcomes for society [126].

Assessing fairness in healthcare intervention prioritization requires various methods and tools to evaluate the distribution of resources and the impact of interventions across different population groups. One commonly used approach is equity analysis, which involves assessing the distribution of healthcare resources and services according to demographic, socioeconomic, and geographic factors to identify disparities and inequities. This effort may include analyzing data on access to healthcare facilities, utilization rates, and health outcomes among different population groups. Additionally, qualitative methods such as community consultations, focus groups, and stakeholder engagement can provide valuable insights into the perceptions and experiences of marginalized communities regarding fairness in healthcare prioritization [127]. Furthermore, health equity impact assessments (HEIAs) can be used to systematically evaluate the potential impact of proposed interventions on health equity and identify strategies to mitigate any negative effects on vulnerable populations [128]. In Indonesia, efforts to promote fairness in healthcare prioritization have included initiatives like the JKN program, aimed at expanding healthcare access and reducing financial barriers for disadvantaged populations. These efforts align with global practices such as the Accountability for Reasonableness (AFR) framework, which promotes fair priority-setting through principles of relevance, publicity, revisions, and enforcement [129].

## Strategies for prioritizing health interventions

**Fig. 3:**
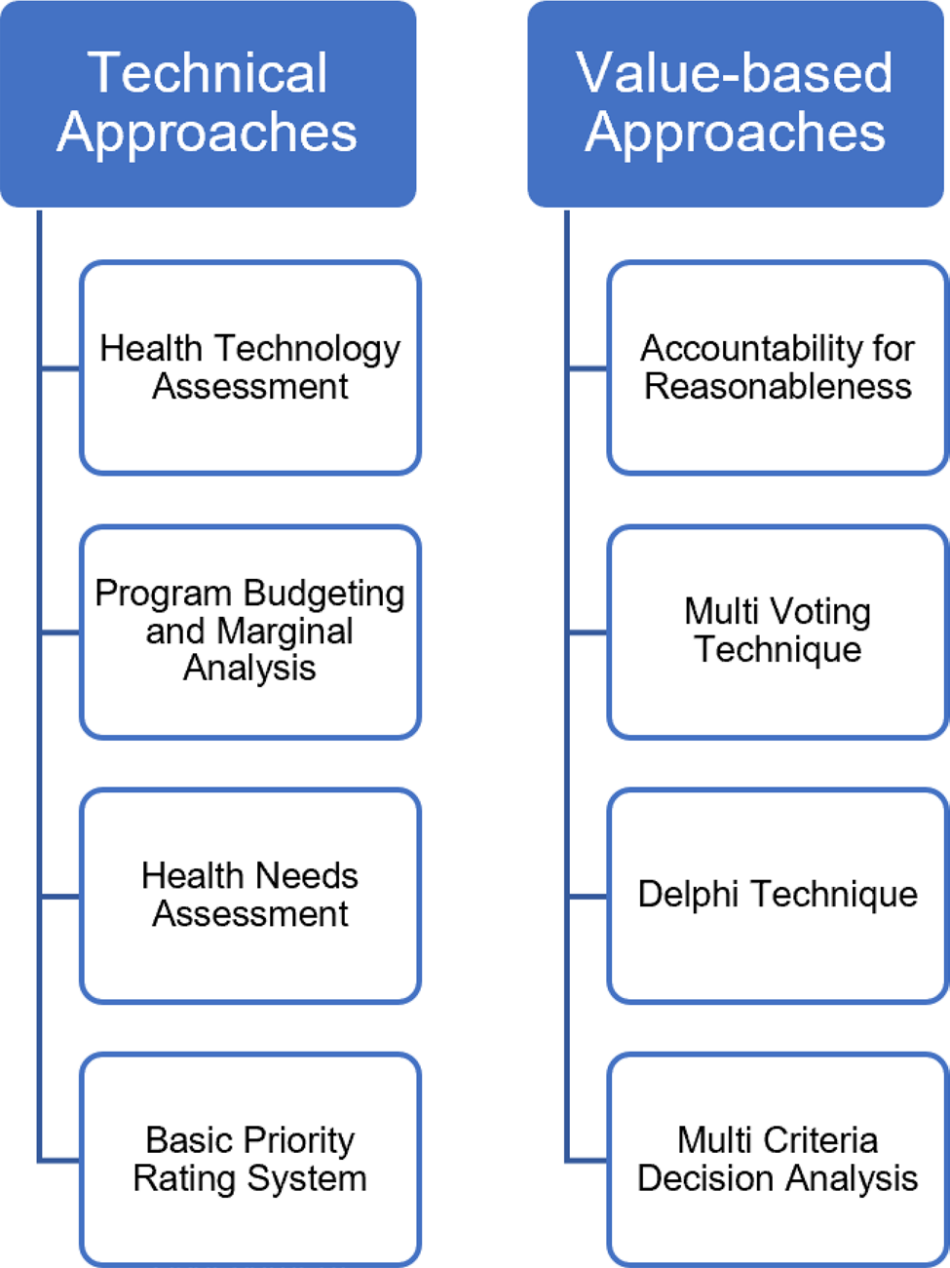
Health intervention prioritization strategies

Decision-makers often rely on various methodologies to navigate the intricate balance between competing health needs and limited resources. The technical and value-based approaches are two primary frameworks for assessing and prioritizing health interventions (Fig. 3) [28, 130]. Within technical approaches, a spectrum of methodologies, ranging from CEA to burden of disease assessments, provides quantitative insights into the efficiency and impact of interventions [131–134]. Meanwhile, value-based approaches explore into the ethical and societal considerations underlying healthcare prioritization, weighing factors such as equity, fairness, and community preferences [135–139]. In practice, these approaches are not strictly separate but rather complementary, often integrated within decision-making frameworks. Multi-Criteria Decision Analysis (MCDA), for instance, combines both technical efficiency measures and ethical priorities to support transparent and inclusive decision-making [140].

In Indonesia, healthcare priority setting involves a mix of technical and value-based approaches, though the extent of their application remains an area for further exploration. Technical methods such as HTA, and Programme Budgeting and Marginal Analysis (PBMA) have been used to provide a structured, evidence-based framework for evaluating interventions, particularly within the JKN system [141, 142]. Meanwhile, value-based approaches, including AFR, the Delphi Technique, and MCDA, are less systematically implemented but offer important considerations of equity, fairness, and stakeholder engagement [141, 143]. Understanding how other approaches are currently utilized and identifying opportunities for a more balanced and transparent framework could strengthen Indonesia’s healthcare prioritization efforts.

### Technical approaches

#### Health technology assessment (HTA)

HTA is a multidisciplinary approach to policy analysis that explores the medical, social, ethical, and economic dimensions of health technology’s development, diffusion, and utilization [144]. HTA aims to comprehensively evaluate health technologies and offer insights to inform decision-making regarding integrating these technologies into healthcare services [145]. A recent study highlighted that the introduction of HTA in Indonesia is still at an early stage, focusing primarily on informing decisions related to basic benefit packages and reimbursement, requiring continuous improvement to effectively address the evolving healthcare landscape [146]. Prioritizing health programs necessitates conducting separate HTA studies for each program [147]. For instance, in breast cancer control programs in Indonesia, distinct HTA investigations are essential for prevention, screening, early detection, diagnostics, therapy, and palliative care interventions [148, 149]. This approach is crucial because while certain screening strategies may yield cost savings, specific palliative care technologies might prove ineffective [150].

HTA processes typically commence either after regulatory approval or concurrently with it. These processes exhibit variations across jurisdictions concerning governance structure, scope (advisory or decision-making), connections to reimbursement mechanisms, and the authority to oversee and modify decisions post-implementation. However, despite these jurisdictional discrepancies, all HTA processes consist of two fundamental phases: evidence assessment, synthesis and appraisal [151, 152]. During the evidence assessment phase, various methodologies can be used. One approach, known as Adaptive HTA, emphasizes flexibility and adaptability to evolving evidence and stakeholder input [153]. This iterative process allows for ongoing assessment and adjustment of decisions based on emerging data, making it particularly suitable for situations with uncertainty or rapidly changing evidence, such as with novel treatments or technologies [154]. In contrast, Full HTA refers to a comprehensive evaluation that typically follows a structured and systematic approach. It involves assessing multiple aspects, including clinical effectiveness, safety, cost-effectiveness, ethical considerations, and broader societal impacts. Full HTA utilizes rigorous methods, such as systematic reviews, economic modeling, and stakeholder engagement, to provide policymakers and healthcare providers with a robust evidence base for informed decision-making regarding the adoption, reimbursement, or use of the technology [155].

The synthesis phase serves as a critical intermediary between evidence assessment and appraisal [156]. In this phase, data from various sources are integrated and contextualized to form a coherent and comprehensive body of evidence [157]. This step requires expertise in systematic review methodologies, data interpretation, and comparative effectiveness analysis [158]. Synthesis ensures that decision-makers receive a balanced, well-structured summary of findings, highlighting uncertainties and limitations while aligning with local healthcare priorities and policy objectives [146].

In the appraisal phase, the outcomes of the evidence assessment undergo review and deliberation by a designated panel or body entrusted with making recommendations regarding payer adoption decisions. Various factors such as equity considerations, acceptability, and input from key stakeholders may be taken into account during this phase. The recommendations typically fall into three categories: (1) positive, (2) positive with restrictions, or (3) negative. Positive restrictions may involve suggestions for price reductions to attain an acceptable value or to meet budget constraints, as well as clinical conditions that delineate access limitations for specific patient subgroups [159].

#### Program budgeting and marginal analysis (PBMA)

PBMA is a prioritization approach in health organizations. It is grounded in principles akin to economic evaluation but noted for its practicality and applicability across various organizational levels [160]. It can be utilized within individual care programs, across multiple programs within a service area, or more broadly across major service areas. It is beneficial for making funding decisions regarding new technologies or drug formularies [160, 161]. The approach is based on two fundamental economic principles: opportunity cost and marginal analysis [162]. Opportunity cost refers to the benefits lost from not choosing the subsequent best alternative use of resources, emphasizing the need to maximize benefits while minimizing costs [162]. Marginal analysis focuses on changes, assessing the costs and benefits of reallocating resources between different areas to determine if such changes are warranted [162]. Both activities can assist decision-makers in identifying progress in implementing health programs with planned budgets and maximizing the benefits of those programs for the population [163].

The PBMA process can be a valuable tool for addressing Indonesia’s healthcare challenges, particularly in optimizing resource allocation within the JKN. For example, Indonesia faces a stark disparity in healthcare access between urban and rural areas, where remote regions struggle with limited healthcare infrastructure and a shortage of medical professionals [164]. Through PBMA, policymakers can prioritize expanding telemedicine programs, such as those initiated by the MOH during the COVID-19 pandemic, to improve access to specialist consultations in rural areas [165]. Similarly, given the rising burden of NCDs like diabetes and hypertension, PBMA can help shift funding toward preventive care, such as community-based screening programs and lifestyle intervention initiatives, which have been piloted in provinces like West Java [166]. However, to ensure this approach’s successful implementation of, Indonesian policymakers must invest in capacity-building initiatives and stakeholder engagement, fostering collaboration and transparency in decision-making processes [167]. Additionally, integrating PBMA with Indonesia’s health information system (e.g., *Satu Sehat*) can facilitate real-time monitoring and evaluation, ensuring that healthcare priorities remain responsive to emerging threats like infectious disease outbreaks and demographic changes [131].

#### Health needs assessment (HNA)

HNA serves as a foundational tool for Indonesian health planners and policymakers, providing a structured approach to identifying and prioritizing healthcare interventions tailored to the country’s unique challenges [168]. Given Indonesia’s diverse geography and healthcare disparities, HNA helps map regional variations in disease patterns, such as the higher prevalence of tuberculosis in densely populated urban centers like Jakarta compared to lower but persistent cases in rural eastern provinces [169, 170]. Therefore, the analysis results enable the Indonesian government to understand the health needs and priorities in each area, which significantly assists in efforts to meet needs accurately and utilize existing resources effectively and efficiently [171]. Unlike economic evaluations that focus on health issues with cost-saving solutions, the mapping results from HNA emphasize the evaluation of fundamental health problems, such as health issues with high mortality rates, where interventions may not be the most cost-effective [172].

According to Nicholas et al. [173], the HNA process involves several critical stages. Initially, careful selection of the target population is essential, considering specific clinical or demographic characteristics and ensuring justification for focus to avoid duplicating efforts. Stakeholder engagement plays a pivotal role in identifying and prioritizing problems the chosen population faces, drawing insights from healthcare professionals, local authorities, and community input. Issues are then assessed and prioritized based on factors such as scale, severity, and intervention feasibility, accounting for both short-term and long-term impacts. Subsequently, action plans are formulated to address identified priorities, guided by stakeholder expertise and evidence from previous successful interventions. These plans entail setting clear objectives, defining responsibilities, and allocating necessary resources, with ongoing evaluation to measure effectiveness and inform future planning endeavors.

#### Basic priority rating system (BPRS)

The BPRS emerges as a valuable tool for Indonesian policymakers and healthcare stakeholders seeking to prioritize health interventions based on priority rankings [174]. BPRS offers a structured approach that balances multiple considerations, including Indonesia’s specific disease burden, intervention effectiveness, cost, acceptability, and fairness, aligning with the WHO’s priority-setting criteria [175]. Thus, BPRS empowers Indonesian decision-makers to allocate resources efficiently, maximize health impact, and address the most pressing needs of populations within the broader context of health priority setting and UHC initiatives. One advantage of this method is that priority setting becomes the explicit objective and be as close as possible based on existing baseline data and resulting numerical values. Health programs are ranked according to their total score, with the program obtaining the highest score being prioritized over others, while those with lower scores receive less priority [176].

According to Douglas et al. [177], the latest version of the BPRS (Hanlon & Pickett, 1984) comprises four key components: (A) assessing the size of the problem (rated on a scale of 0–10 points); (B) evaluating the seriousness of the problem (rated on a scale of 0–20 points); (C) measuring effectiveness (rated on a scale of 0–10 points); and (D) considering propriety, economics, acceptability, resources, and legality (PEARL), which are assigned a score of either 0 or 1. Following the scoring of each health problem against these criteria, the corresponding values are applied in the following equation to derive a total score:

$${{\left( {A + B} \right)C} \over 3}\,\times\,D$$

### Value-based approaches

#### Accountability for reasonableness (AFR)

AFR is an ethics-based approach in the priority-setting process that aims to ensure legitimacy and fairness to gain support from all stakeholders involved in implementing a health program or intervention [178]. At its core, AFR seeks to promote fairness and transparency in decision-making processes related to resource allocation [179]. The framework emphasizes four key conditions guiding priority-setting activities: *relevance, publicity, revisability, and enforcement (Textbox 1)* [180]. Within the AFR framework, decision-makers are encouraged to engage stakeholders representing diverse perspectives, including healthcare providers, patients, policymakers, and community members, in a participatory process [181]. By fostering a culture of accountability and transparency, AFR aims to mitigate potential conflicts and disparities in healthcare resource allocation, ultimately contributing to more equitable and just health systems [182].

In practice, AFR has been applied in various settings in Indonesia to guide priority-setting efforts in healthcare, such as budget allocation within the JKN, evaluating the fairness of infectious disease interventions, and ensuring the equitable distribution of healthcare services in underserved regions like West Java [143, 183]. While AFR provides a valuable ethical framework for navigating complex priority-setting decisions, its successful implementation hinges on strong leadership, effective communication, and ongoing stakeholder engagement [184]. By embracing the principles of AFR, policymakers and healthcare leaders in Indonesia can work towards fostering more significant equity, transparency, and accountability in allocating healthcare resources, ultimately advancing the goal of achieving UHC and improving health outcomes for all Indonesians [182, 185].

**Textbox 1:** Principles for ensuring a fair process

• Relevance: The rationale behind priority-setting decisions should be based on evidence and principles that reasonable individuals can agree are pertinent to the context. This situation fosters cooperation among “fair-minded” individuals who seek terms justifiable to each other, thus narrowing the scope of controversy. Specifying that reasons must be relevant to the specific priority-setting context further narrows potential disputes.
• Publicity: Priority-setting decisions and their underlying rationales must be made publicly accessible, emphasizing the process transparency. In matters concerning people’s well-being, justice cannot tolerate secrecy.
• Revisions/Appeals: Mechanisms for challenging decisions must be in place, providing stakeholders with the opportunity to raise considerations and seek revisions based on valid concerns.
• Enforcement: The process should be regulated by voluntary or public means to ensure compliance with the first three conditions.

#### Multi voting technique (MVT)

MVT, also known as the Nominal Group Technique (NGT), is participatory in health-priority settings [186]. It aims to aggregate individual preferences and opinions into collective decisions within a facilitated group setting, often working with informant panels to prioritize responses to specific questions [187]. MVT provides clear and concise documentation of participants’ responses, reducing the risk of investigator-induced interpretive bias while ensuring equitable participation rates [188]. Furthermore, it assigns equal weight to the input from all participants through anonymously ranked responses, assumed to represent the collective viewpoints of the group participants [188].

In MVT, Indonesian stakeholders are given a list of health interventions or priorities and asked to rank or vote for their top choices individually, allowing for a transparent and participatory approach to decision-making [189]. Each participant typically receives a predetermined number of votes, which they can allocate to their preferred options. Once all votes are cast, the votes are tallied, and the priorities with the highest number of votes are identified as the group’s collective preferences [190]. MVT offers several practical advantages in the Indonesian context. Firstly, it is time-efficient [191], conducted in a single session yet yielded significant information, which is ideal for busy hospital clinicians [192]. Secondly, it is cost-effective, often requiring minimal expenditure, with venues provided at no cost and refreshments covered by grants, fostering a conducive atmosphere [193]. Thirdly, it demands little preparation from participants, ensuring accessibility even for senior clinicians with demanding schedules [194]. Lastly, NGT enables immediate dissemination of results within the session, enhancing participant satisfaction by providing a sense of accomplishment [195].

#### Delphi Technique

The Delphi Technique is a structured and iterative method for gathering and refining expert opinions to reach consensus on complex issues, such as health intervention prioritization [196]. It involves multiple rounds of anonymous surveys or questionnaires administered to a panel of experts with diverse backgrounds and expertise relevant to the topic under consideration [197]. The first round of the process involves opinion exploration, during which the facilitator sends several questions to the experts regarding the issue to be discussed, whether conveyed in writing (letters or emails) or orally (telephone), with experts asked to provide comprehensive answers and send them back to the facilitator [198]. In the second round, the facilitator summarizes the experts’ opinions and communicates them back to all participants, allowing each expert to review the opinions of others, maintain or revise their own opinions accordingly, and then send them back to the facilitator [199]. The third round focuses on understanding the reasoning behind any persisting disagreements, helping to refine and align perspectives [200]. The final round is evaluation, where this process continues until the investigative team is confident that all opinions are the result of mature thinking [201].

Prioritizing health interventions in Indonesia requires a systematic and inclusive approach that considers multiple criteria and engages key stakeholders. The Delphi Technique offers numerous advantages. Firstly, it enables the consultation of a larger number of individuals without the constraints of face-to-face meetings, accommodating busy schedules and ensuring broader participation [202]. Secondly, it provides access to specialized expertise from a diverse array of experts on specific topics without necessitating significant human and financial resources [203]. Additionally, participants can contribute at their convenience, fostering inclusivity and ensuring a comprehensive range of considered perspectives [204]. Furthermore, this approach allows for iterative discussions around the topic in question, facilitating deeper exploration and refinement of ideas over time [205].

#### Multi criteria decision analysis (MCDA)

MCDA is a robust method to evaluate and prioritize health interventions, combining both technical and value-based methodologies [206]. MCDA enables establishing a comprehensive set of criteria, the assessment of interventions’ performance based on these criteria within a performance matrix, and subsequent qualitative or quantitative analysis to rank or order the interventions [207]. In prioritizing with this method, five criteria can be applied. First, MCDA focuses on optimizing public health by maximizing health outcomes such as reducing mortality rates, morbidity, disease burden, and improving overall quality of life [208]. Additionally, MCDA considers the equitable distribution of health benefits across different demographic groups to address disparities and promote health equity [209]. Moreover, it incorporates societal preferences by engaging stakeholders to ensure interventions align with community values and priorities, enhancing acceptability and legitimacy [210]. Furthermore, MCDA acknowledges budget constraints and practical considerations, evaluating the cost-effectiveness and feasibility of interventions within available resources [211]. Finally, while aiming for evidence-based decision-making, political considerations such as public opinion, stakeholder interests, and policy priorities may also influence the prioritization process, highlighting the need for transparent and inclusive decision-making [212].

Implementing MCDA in Indonesia can enhance prioritizing health interventions and align decision-making with national health goals and priorities. By systematically considering multiple criteria, including those specific to Indonesia’s healthcare context, such as resource availability, cultural preferences, and population needs, MCDA can facilitate a more informed and equitable allocation of resources [213, 214]. Additionally, the participatory nature of MCDA encourages collaboration among stakeholders, fostering greater transparency and accountability in decision-making processes. Indonesian stakeholders can leverage MCDA to navigate complex trade-offs and identify interventions that offer the most outstanding value in improving population health outcomes and advancing UHC [215].

MCDA has become increasingly influential in healthcare priority setting in Indonesia, providing a structured approach to decision-making that balances both technical and social considerations. Inotai et al. highlighted its application in off-patent drug decision-making, identifying key criteria beyond price, such as manufacturing quality, product equivalence, stability, supply reliability, real-world outcomes, and pharmacovigilance [216]. Another study employed the Country-led Assessment for Prioritization on Immunization (CAPACITI) decision-support tool within an MCDA framework to prioritize new vaccines for inclusion in the National Immunization Program (NIP). This tool has played a crucial role in evaluating vaccines such as Pneumococcal Conjugate Vaccine (PCV), Rotavirus Vaccine (RV), Human Papillomavirus Vaccine (HPV), and Japanese Encephalitis Vaccine (JE), as outlined in Indonesia’s 2020–2024 Comprehensive Multi-Year Plan [217]. Additionally, Suwantika et al. (2021) applied the Strategic Multi-Attribute Ranking Tool for Vaccines (SMART Vaccines) within an MCDA approach to assess vaccine introductions, ranking PCV, rotavirus, HPV, and JE vaccines as priorities [218]. These studies highlight Indonesia’s growing commitment to integrating MCDA into healthcare decision-making.

## Concept of health priorities in UHC

Building upon the discussion of prioritization criteria and strategies, this section explores how healthcare priority setting is integrated into Indonesia’s broader efforts to achieve UHC. The aim is to demonstrate how the principles and frameworks discussed earlier are reflected in real-world policy decisions related to service coverage, financing, and access. This section also highlights key challenges that shape the operationalization of health priorities, including trade-offs in benefit package design, regional inequities, and limitations in healthcare workforce capacity. Exploring these factors affirms the essential role of priority setting in driving equitable and sustainable healthcare reforms.

### Health priority indicators for advancing UHC

UHC refers to a healthcare system where all individuals in a particular country or region have access to quality health services they need without facing financial hardship or impoverishment [219]. UHC plays a fundamental role in alleviating poverty and empowering individuals to manage their health, enabling them to contribute to society actively [220]. Hence, it comes as no surprise that UHC was chosen as a critical global health priority within the United Nations Sustainable Development Goals (SDGs) adopted in September 2015 [221]. SDGs aims to realize the overarching health goal (Goal 3) of “ensuring healthy lives and promoting well-being at all ages” [222]. Goal 3.8 specifically aims to “achieve UHC, including financial risk protection, access to quality essential health-care services and access to safe, effective, quality and affordable essential medicines and vaccines for all” [223]. Moreover, UHC commands significant attention in the Declaration for Transforming Our World, endorsed by heads of government preceding the identification of the SDGs, as it aligns with the fundamental commitment within the SDGs to leave no one behind: “To promote physical and mental health and well-being, and to extend life expectancy for all, we must achieve UHC and access to quality health care. No one must be left behind” [224].

To achieve this, the government should focus on three main areas: broadening the scope of services funded through pooled resources, reducing reliance on out-of-pocket payments, and increasing population coverage for both service access and financial protection against healthcare costs [225, 226]. Achieving expanded services for unreached population segments entails prioritizing vulnerable and marginalized groups, while maximizing service provision involves enhancing the efficiency of service packages, and enhancing financial risk protection necessitates fee reduction, especially for poor and vulnerable populations [227–229]. The WHO identifies various indicators of Healthcare Service Coverage (HSC) within a country, encompassing reproductive and newborn health (including family planning, antenatal care, and skilled birth attendance), child immunization (such as three doses of the diphtheria-pertussis-tetanus vaccine or DTP), treatment for infectious diseases (including antiretroviral therapy and tuberculosis), and non-health sector determinants (such as improvements in water sources and sanitation facilities) [230, 231]. These indicators can serve as references in decision-making regarding prioritizing health programs and contributing to efforts to achieve UHC from the government’s perspective. Establishing health priorities in implementing UHC in Indonesia involves a political decision-making process incorporating public information and stakeholder negotiations [232]. One challenge in supporting UHC reform is the diverse perceptions among policymakers, some of whom view UHC solely as a conceptual framework, emphasizing progress that is challenging to measure. UHC with its coverage dimensions, including healthcare services, financial protection, and population coverage, can constitute a continuous dynamic process, responding to changes in demographic, epidemiological, technological trends, and societal expectations [233]. Despite persistent challenges of healthcare service inequality and significant poverty levels resulting from healthcare expenditures, measurable progress towards UHC in Indonesia is evident [234].

### Health priorities implementation for UHC in LMICs contexts: Indonesia as a reference case

The successful implementation of health priorities for UHC in LMICs is a critical endeavor, requiring a concerted effort to translate prioritized health interventions into tangible improvements in population health and well-being [235]. This process is inherently complex, marked by challenges such as limited financial resources, weak health systems, and competing health agendas [236]. Moreover, LMICs grapple with additional dynamics, including epidemiological transitions, economic growth, rising healthcare costs, and reductions in international health aid [237, 238]. In response, many countries emphasize the pivotal role of healthcare workers. Ensuring alignment between the population’s healthcare needs and the availability of skilled and adequately trained healthcare professionals is paramount for accelerating progress toward UHC [239]. Indeed, human resources emerge as a linchpin in the implementation journey of UHC, with the success of nations hinging on policies aimed at expanding population coverage and improving health outcomes through the strategic deployment and support of healthcare workers [240, 241]. UHC is being implemented by many countries around the world, including Indonesia, with various timelines for implementation, especially with the recent COVID-19 pandemic, which has brought disruption to various sectors, one of which is the health insurance system [242].

Indonesia has a significantly smaller healthcare workforce compared to neighboring countries like Singapore and Malaysia, a challenge worsened by the toll of the COVID-19 pandemic on healthcare worker deaths [243]. To advance UHC, Indonesia introduced the National Health Insurance program JKN that has been implemented and managed through the BPJS Kesehatan since January 1, 2014, covering approximately 226.7 million individuals or about 83% of Indonesia’s population [242, 244]. While this initiative reflects the government’s commitment to UHC, its implementation varies across cities and districts due to regional policy differences. Ensuring the effective delivery of prioritized health interventions remains a challenge, as financial constraints necessitate continuous adjustments to benefit packages, reimbursement structures, and service delivery strategies [245].

One of the biggest challenges Indonesia faces in implementing JKN is making difficult trade-offs to sustain the system while expanding coverage [246]. Rising healthcare costs, an increasing burden of NCDs, and growing demand for advanced treatments put significant pressure on the program’s financial stability [247]. To manage expenditures, BPJS Kesehatan has adjusted reimbursement policies, utilizing capitation for primary care and case-based payments (INA-CBGs) for hospital services [248]. Another critical trade-off involves decisions on which treatments and services should be fully covered, partially subsidized, or excluded, requiring careful consideration of cost-effectiveness, disease burden, and equity. To support these difficult decisions, Indonesia has integrated HTA into its priority-setting process. The Indonesia Health Technology Assessment Committee (InaHTAC) evaluates medical interventions for their inclusion in JKNs benefits package, helping policymakers determine which treatments provide the best value for money [36]. However, implementing HTA remains challenging due to limited institutional capacity, data constraints, and disparities in healthcare infrastructure across regions [249]. Strengthening HTA processes and integrating them more effectively into policy decisions are essential for ensuring that JKN can allocate resources efficiently.

Multi-stakeholder collaboration is another key aspect of Indonesia’s healthcare priority-setting. Decision-making involves the MOH, BPJS, local governments, healthcare providers, and civil society organizations [250]. However, Indonesia’s decentralized governance structure creates disparities in JKN implementation, as each region has varying capacities to deliver healthcare services [251]. While regional autonomy allows for flexibility in addressing local needs, it also leads to inconsistencies in service quality, resource distribution, and policy execution. Improving coordination, enhancing data-sharing mechanisms, and strengthening regulatory oversight are necessary steps to ensure a more equitable and efficient system [252]. Moreover, Indonesia can draw valuable insights from the health priority implementation strategies employed by Brazil and Thailand, which offer useful reference points in its pursuit of UHC.

Brazil has made significant strides towards achieving UHC by establishing the Sistema Único de Saúde (SUS) in 1988, an integrated healthcare system focused on community services and improving access for underserved populations [253, 254]. Preceding the launch of SUS, the Brazilian government implemented a capacity-building program for training and managing human resources in the healthcare sector [255]. Between 1987 and 2009, this program was fortified to enhance community acceptance, service quality, and address the gap between the availability and demand for healthcare workers, particularly in primary care. These efforts led to a notable increase healthcare workers, with a 500% rise in nurses and a 66% increase in doctors during this period [256]. From 2002 to 2012, the number of family health teams doubled from 15,000 to 30,000, and by 2013, basic health unit accessibility reached 57% of the population [257]. Consequently, there was a substantial decline in neonatal mortality rates from 26.8 to 9.7 per 1,000 live births and infant mortality rates from 58 to 15.6 per 1,000 live births over the same period [256].

Similarly, Thailand has pursued UHC by implementing various policies to bolster the capacity and caliber of its human resources. The period from 1990 to 2009 is a testament to the achievements that have significantly contributed to Thailand’s UHC success [258]. Since the 1970s, the country has adopted substantial policies to facilitate providing and financing healthcare services for the impoverished [259]. In 1995, introducing basic healthcare services at the district level, enabled by comprehensive healthcare workforce policies, marked a pivotal moment [260]. Subsequently, Thailand prioritized continual reflection, improvement, and the elevation of healthcare worker quality through the development of professional councils, standardization of curricula, and the implementation of new licensing and re-licensing procedures for healthcare professionals [261]. Notably, between 1991 and 2009, there was a remarkable surge in the number of healthcare workers in Thailand, with a 210% increase in nurses and a 186% rise in doctors, outpacing the population growth rate of just 13% [256]. This substantial expansion of the healthcare workforce significantly enhanced community accessibility to healthcare services, thereby propelling Thailand’s progress toward achieving UHC [262].

The experiences of Brazil and Thailand highlight the importance of prioritizing human resources within the healthcare sector to achieve UHC, emphasizing clear and targeted policies backed by strong partnerships between various stakeholders [263, 264]. Therefore, Indonesia’s success in enhancing healthcare worker capacity and quality depends on robust political leadership and stakeholder commitment, supported by consistent legislation and regulations [265]. Additionally, a comprehensive strategy is essential, encompassing multiple dimensions related to efforts aimed at improving service quality and enhancing healthcare worker outreach, including availability (supply and production), accessibility (spatial, temporal, and financial), acceptability (gender, social, and cultural), and quality (competence and regulation) [266, 267]. This would align with the vision of “*Indonesia Maju*”, which emphasizes superior human resources in the healthcare field, more productivity, and competitiveness to enhance the quality of healthcare services for the population [268, 269].

## Conclusion

In conclusion, while Indonesia has made significant progress in healthcare priority setting, there remain substantial opportunities for improvement. The integration of key criteria such as disease burden, intervention effectiveness, cost, acceptability, and fairness, as well as a combination of technical and value-based methodologies has laid the groundwork for more equitable decision-making. However, persistent challenges hinder the full realization of these efforts. One critical issue is the absence of a fully consolidated and institutionalized national framework for healthcare prioritization. This structural gap contributes to inconsistent implementation across regions and limits the effective use of available tools and criteria. Additionally, disparities in healthcare infrastructure, regional governance capacity, and data quality further complicate the consistent application of prioritization strategies. Moving forward, it is essential to strengthen institutional coordination, invest in national-level policy integration, and ensure stakeholder engagement in the design and implementation of priority-setting processes. Addressing ethical trade-offs and improving the transparency of decision-making will also be crucial. While the Indonesian experience offers context-specific lessons, its applicability as a general model for other LMICs may be limited. Instead, it should be viewed as a case study illustrating both the progress and the ongoing complexities of healthcare prioritization in a decentralized, resource-constrained setting.

## Data Availability

This review is based on data and materials available in the public domain and from published literature. All sources of data and materials are cited appropriately in the manuscript. No new datasets were generated during the course of this study.

## Abbreviations

AFR: Accountability for Reasonableness
BIA: Budget Impact Analysis
BPRS: Basic Priority Rating System
BPJS: Badan Penyelenggara Jaminan Sosial Kesehatan
CEA: Cost-effectiveness analysis
DALE: Disability-Adjusted Life Expectancy
DFLE: Disability-Free Life Expectancy
DTP: Diphtheria-pertussis-tetanus
GBD: Global Burden of Disease
HEIAs: Health equity impact assessments
HITAP: Thailand’s Health Intervention and Technology Assessment Program
HLY: Healthy Life Years
HNA: Health Needs Assessment
HTA: Health Technology Assessment
HSC: Healthcare Service Coverage
HSUVs: Health state utility values
JKN: National health insurance scheme
LMICs: Low- and middle-income countries
MCDA: Multi Criteria Decision Analysis
MOH: Ministry of Health
MR: Measles-Rubella
MUI: Indonesian Ulema Council
MVT: Multi Voting Technique
NCDs: Non-communicable diseases
NGT: Nominal Group Technique
NIP: National Immunization Program
PBMA: Program Budgeting and Marginal Analysis
PEARL: Propriety, economics, acceptability, resources, and legality
QALYs: Quality-adjusted life years
Renstra: Strategic Plans
SDGs: Sustainable Development Goals
SMPH: Summary measures of population health
SUS: Sistema Único de Saúde
TFA: Theoretical Framework of Acceptability
UHC: Universal Health Coverage
WHO: World Health Organization
YLDs: Years Lived with Disability
YLLs: Years of Life Lost
